# High Gingival Bleeding Burden in Late Pregnancy Is Associated with a Salivary Profile of Inflammatory, Regulatory, and Stress-Response Signals

**DOI:** 10.3390/life16091423

**Published:** 2026-08-27

**Authors:** Gerson Aparecido Foratori-Júnior, Hélvis E. S. Paz, Roosevelt Da Silva Bastos, Luiz Renato Paranhos, Nagihan Bostanci, Marília Afonso Rabelo Buzalaf, Guy Howard Carpenter

**Affiliations:** 1 Department of Biological Sciences, Bauru School of Dentistry, University of São Paulo, Bauru 17012-901, Brazil; 2 Centre for Host-Microbiome Interactions, Faculty of Dental, Oral & Craniofacial Sciences, King’s College London, London SE1 1UL, UK; 3 Division of Periodontology, Department of Dental Clinics, Oral Pathology and Oral Surgery, School of Dentistry, Federal University of Minas Gerais, Belo Horizonte 31270-901, Brazil; 4 Department of Pediatric Dentistry, Orthodontics and Public Health, Bauru School of Dentistry, University of São Paulo, Bauru 17012-901, Brazil; 5 Department of Orthodontics, School of Dentistry, Federal University of Uberlândia, Uberlandia 38405-320, Brazil; 6 Division of Oral Health and Periodontology, Department of Dental Medicine, Karolinska Institutet, 14152 Stockholm, Sweden

**Keywords:** biomarkers, cytokines, gingivitis, pregnancy, saliva

## Abstract

Pregnancy increases susceptibility to gingival inflammation, and saliva may help characterize associated host-response profiles. This cross-sectional study compared nine salivary proteins and inflammatory mediators between pregnant women with high and low gingival bleeding burdens. Twenty-four women were classified by bleeding on probing (BOP) into G1 (BOP ≥ 50%; *n* = 12) and G2 (BOP < 30%; *n* = 12). Unstimulated saliva was collected, and RBP4, β2-microglobulin, clusterin, cystatin C, IL-1β, IL-6, IL-8, IL-10, and TNF-α were quantified using multiplex bead-based immunoassays. Primary comparisons used ANCOVA adjusted for maternal age and total protein concentration, with additional adjustment for visible biofilm in sensitivity analyses. In the primary adjusted analyses, G1 showed higher RBP4 (FDR-adjusted *p* = 0.004; partial η^2^ = 0.466) and clusterin (FDR-adjusted *p* = 0.006; partial η^2^ = 0.413), and lower IL-10 (FDR-adjusted *p* = 0.032; partial η^2^ = 0.285). In the sensitivity analyses, significant group effects were observed for RBP4 (FDR-adjusted *p* = 0.026; partial η^2^ = 0.381) and IL-8 (FDR-adjusted *p* = 0.044; partial η^2^ = 0.303). These findings demonstrate associations between high gingival bleeding burden and a salivary profile involving inflammatory/chemotactic, regulatory, and stress-response signals.

## 1. Introduction

Periodontitis is a chronic inflammatory disease of the periodontium characterized by a dysregulated host immune response to a dysbiotic subgingival biofilm [1,2]. Although microbial biofilm initiates the disease, periodontal breakdown is largely driven by an imbalance between microbial challenge and host defense, leading to damage to the tissues responsible for anchoring the teeth, including the periodontal ligament and alveolar bone, and potentially culminating in tooth loss if left untreated [1,2]. The accumulation of bacterial biofilm along the gingival margin first induces biofilm-induced gingivitis, a local inflammatory condition that remains fully reversible at this early stage [3,4]. Although biofilm-induced gingivitis is reversible, persistent inflammation in susceptible individuals may coexist with conditions that favor the irreversible destruction of the periodontal support apparatus [1,3].

Pregnancy is a period in which gingival tissues are particularly susceptible to inflammatory changes. During gestation, marked changes in estrogen and progesterone levels can increase vascular permeability and modulate cellular and humoral immune responses in gingival tissues, thereby amplifying biofilm-induced inflammation [5]. Pregnancy has also been associated with alterations in the salivary microbiome, including reduced microbial richness and enrichment of taxa associated with periodontal inflammation, which may interact with the heightened gingival inflammatory response observed during this period [6]. Pregnancy-associated gingivitis, reported to affect 30% to 100% of pregnant women depending on the population studied, typically manifests as erythematous, edematous gingiva that bleeds readily upon brushing, flossing, or periodontal probing [5]. Although these changes are generally transient and may resolve after delivery when adequate oral hygiene is maintained, a high bleeding burden during pregnancy may reflect a more pronounced host inflammatory response rather than merely a localized clinical finding [7,8]. This is particularly relevant because gingival inflammation and periodontal diseases during pregnancy have been discussed in relation to maternal systemic inflammatory status and adverse gestational conditions, even though their clinical implications differ according to disease severity and periodontal attachment involvement [9,10]. Moreover, emerging evidence suggests that maternal oral inflammatory conditions may influence early microbial colonization patterns in offspring [11,12]. Therefore, investigating salivary biomarkers associated with contrasting levels of gingival bleeding in pregnant women may help characterize host-response profiles in the absence of destructive periodontal involvement.

Despite its clinical relevance, the recognition and monitoring of gingival inflammation during pregnancy are not always straightforward. Pregnant women are known to attend dental appointments less frequently than the general population, a pattern attributed to insufficient oral health education, restricted access to care, persistent myths and misconceptions about dental treatment during pregnancy, and inconsistent or outdated guidance from some healthcare professionals regarding its safety [13,14]. In addition, gestational gingival inflammation may negatively affect women’s daily well-being, as oral inflammatory conditions during pregnancy have been associated with poorer oral health-related quality of life [15]. These challenges are important because bleeding on probing, although clinically simple and widely used, captures only one aspect of a broader host–biofilm inflammatory interaction. Therefore, complementary non-invasive approaches that help characterize the biological profile underlying different degrees of gingival bleeding may improve our understanding of pregnancy-associated gingival inflammation. In this context, saliva is a useful biological matrix, as it can be collected easily and repeatedly during pregnancy and may reflect local immune–inflammatory responses occurring within the oral cavity.

At the molecular level, salivary biomarkers can help characterize host-response patterns associated with pregnancy-related gingival inflammation. Gingival bleeding reflects the clinical expression of inflammatory processes involving biofilm-induced cytokine release, neutrophil recruitment, vascular changes, regulatory immune responses, and tissue remodeling. Thus, the assessment of a targeted panel of salivary mediators may help identify biological differences that are not fully captured by bleeding on probing alone. Previous studies have shown that saliva can reflect oral inflammatory and microbial shifts associated with periodontal conditions and treatment response [16,17], while salivary cytokines and antimicrobial proteins have been associated with pregnancy-induced periodontal inflammation [18]. In pregnant women, salivary IL-6 and TNF-α have also been investigated according to periodontal status [19], and recent proteomic studies have shown that generalized gingivitis and excessive gingival bleeding during pregnancy are associated with distinct salivary protein signatures involving immune, antimicrobial, and tissue-protective pathways [20,21]. In this context, IL-1β, TNF-α, and IL-6 represent key mediators of pro-inflammatory activation, whereas IL-8 is particularly relevant to neutrophil chemotaxis and innate immune responses at biofilm-challenged sites [22,23]. IL-10 provides a regulatory counterpoint by limiting excessive cytokine production and tissue-damaging inflammation [24]. Beyond these classic cytokines, RBP4, β2-microglobulin, clusterin, and cystatin C may offer complementary information on metabolic–inflammatory crosstalk, periodontal immune activation, oxidative and inflammatory stress responses, and protease regulation during periodontal tissue remodeling [25,26,27,28].

However, evidence regarding this targeted salivary panel in pregnant women with contrasting gingival bleeding burdens remains limited, particularly in clinically well-defined samples without periodontitis. Therefore, this study aimed to compare salivary proteins and inflammatory mediators between third-trimester pregnant women with high and low gingival bleeding burdens. We hypothesized that women with high gingival bleeding burden would exhibit a distinct salivary profile involving inflammatory, regulatory, and stress-response signals.

## 2. Methods

### 2.1. Ethical Considerations

This study was conducted in accordance with the Declaration of Helsinki and was approved by the Research Ethics Committee of the Bauru School of Dentistry, University of São Paulo (CAAE 77660724.3.0000.5417, approved on 6 March 2024). All participants provided written informed consent.

### 2.2. Study Design and Participants

This observational, cross-sectional, and analytical study was reported in accordance with the Strengthening the Reporting of Observational Studies in Epidemiology (STROBE) guidelines [29].

Fifty-four pregnant women aged 18–40 years, in the third trimester of pregnancy (≥27 gestational weeks) and receiving regular obstetric care at primary healthcare units in Bauru, São Paulo, Brazil, met the eligibility criteria and underwent periodontal assessment between March 2024 and February 2025. The final analytical sample comprised 24 participants selected according to the gingival bleeding criteria described below. The exclusion criteria were systemic arterial hypertension diagnosed before or during pregnancy (blood pressure ≥ 140/90 mmHg); pre-eclampsia; gestational diabetes mellitus, defined as fasting plasma glucose of 92–125 mg/dL, plasma glucose ≥ 180 mg/dL 1 h after the glucose load, or 153–199 mg/dL 2 h after the glucose load; malnutrition, defined as a pre-pregnancy body mass index (BMI) < 18.5 kg/m^2^; morbid obesity, defined as BMI ≥ 40 kg/m^2^ or BMI ≥ 35 kg/m^2^ in the presence of comorbidities; a medical recommendation for strict bed rest; use during pregnancy of antibiotics or medications capable of affecting periodontal status or salivary flow, including immunosuppressants, anticonvulsants, and calcium channel blockers; low unstimulated salivary flow rate, defined as <0.25 mL/min; ongoing orthodontic or periodontal treatment; use of alcohol, tobacco, or illicit drugs during pregnancy; diagnosed periodontitis, defined as detectable interdental clinical attachment loss at two or more non-adjacent teeth, or buccal or oral clinical attachment loss ≥ 3 mm with probing depth > 3 mm at two or more teeth, provided that the attachment loss was not attributable to non-periodontal causes [2]; and extensive tooth loss, defined as more than two missing teeth per hemiarch. Women with a history of periodontal treatment who were undergoing supportive periodontal care and presented with a reduced but stable periodontium resulting from previous attachment loss were also excluded. By contrast, women with gingival recession unrelated to periodontal disease, such as trauma-induced recession, remained eligible because this condition was not expected to bias the study outcomes.

According to the 2018 EFP/AAP classification of periodontal diseases and conditions, generalized gingivitis is defined by BOP at >30% of sites, whereas localized gingivitis encompasses BOP scores from 10% to 30% [30]. This established threshold was retained for diagnostic interpretation and was not intended to be replaced by a new classification. For the analytical purposes of the present study, however, women with BOP values ranging from 30% to <50% were excluded from the group comparison to increase the clinical and biological contrast between groups and reduce potential overlap in salivary inflammatory profiles. Individuals with BOP values close to the conventional 30% threshold may exhibit similar biological profiles despite being assigned to different diagnostic categories, whereas inclusion of the intermediate range could attenuate between-group contrasts and obscure potentially distinct salivary profiles. High gingival bleeding burden was therefore operationally defined as BOP ≥ 50%, in accordance with previous biomolecular studies in pregnant women that used a comparable BOP threshold around 50% [20,21]. All eligible women with BOP ≥ 50% were included in G1, whereas G2 comprised 12 women randomly selected, before laboratory analysis, from all eligible participants with BOP < 30% using a computer-generated random-number sequence. The analytical sample size was constrained by the number of eligible women presenting the high gingival bleeding phenotype during the predefined recruitment period, with an equal number of participants selected for the low gingival bleeding group to ensure balanced group sizes. In the absence of reliable preliminary estimates for the adjusted group effects, no analyte-specific a priori sample size calculation was performed.

### 2.3. Sociodemographic, Systemic, Oral Hygiene, and Periodontal Assessments

Sociodemographic, systemic, anthropometric, and oral hygiene variables were assessed to characterize the study population and examine the comparability of the groups with respect to factors that could influence periodontal inflammation or the interpretation of salivary biomarker concentrations. The variables included maternal age, gravidity, educational level, monthly household income, pre-pregnancy body mass index (BMI), gestational weight gain, daily toothbrushing frequency, and frequency of interdental cleaning with dental floss or interdental brushes. Information available in the medical records maintained by the primary healthcare units was extracted from those records. Variables not documented in the medical records were obtained by participant self-report during a face-to-face interview conducted at the study visit. Educational level was coded from 0 to 6 as follows: 0, no formal education; 1, incomplete primary education; 2, completed primary education; 3, incomplete secondary education; 4, completed secondary education; 5, incomplete higher education; and 6, completed higher education. Monthly household income was categorized according to the Brazilian minimum wage (MW) as follows: 1, up to 1 MW; 2, >1 to 2 MW; 3, >2 to 3 MW; 4, >3 to 4 MW; 5, >4 to 5 MW; and 6, >5 MW. The official monthly MW was BRL 1412.00 in 2024 and BRL 1518.00 in 2025 [15].

Full-mouth periodontal examinations were performed in all erupted permanent teeth, excluding third molars, by a single experienced and calibrated examiner using a standard manual periodontal probe (Hu-Friedy, Chicago, IL, USA). Examiner calibration had been conducted previously in 15 non-pregnant adults. Agreement for classification according to the presence or absence of gingivitis and periodontitis was assessed using Cohen’s kappa coefficient (κ = 0.95), whereas intra-examiner reproducibility for periodontal probing depth was assessed using the intraclass correlation coefficient after repeated examinations performed 15 days apart (ICC = 0.88). Visible biofilm and bleeding on probing (BOP) were recorded dichotomously as present or absent at six sites per tooth (mesiobuccal, mid-buccal, distobuccal, mesiolingual/palatal, mid-lingual/palatal, and distolingual/palatal) and expressed as the percentage of examined sites. Periodontal probing depth (PPD) and clinical attachment level (CAL) were measured in millimeters at the same six sites per tooth. For each participant, mean PPD and mean CAL were calculated by averaging the measurements obtained across all examined sites.

### 2.4. Saliva Collection

Unstimulated whole-mouth saliva was collected before the periodontal examination to avoid contamination with blood resulting from probing-induced gingival bleeding. All samples were collected in the morning, between 9:00 and 11:00 a.m., at least 1 h after the last food or beverage intake [31]. Participants remained seated in an upright position and at rest for 15 min before collection. They then rinsed their mouths with 5 mL of deionized water and expectorated the rinse. Unstimulated whole-mouth saliva was subsequently collected by passive drooling for 10 min into a sterile 50 mL Falcon tube maintained on ice. The total volume collected during this period was recorded for each participant. The unstimulated salivary flow rate was calculated by dividing the collected volume by the 10 min collection period and was expressed in mL/min. Samples were centrifuged at 4500× *g* for 15 min at 4 °C to remove cellular and particulate debris, and the resulting supernatant was aliquoted and stored at −80 °C until analysis [32,33,34].

### 2.5. Quantification of Total Salivary Protein and Multiplex Analysis of Salivary Biomarkers

Before laboratory analysis, the samples were randomly ordered and relabeled with unique identification codes by a researcher not involved in the biomarker assays. The researcher performing the assays remained blinded to gingival bleeding group allocation throughout all laboratory procedures.

Total salivary protein concentration was quantified in saliva supernatants using the Bradford colorimetric assay (Bio-Rad Laboratories, Hercules, CA, USA), according to the manufacturer’s instructions. A 1 µL aliquot of each sample was analyzed in duplicate, and the mean of the two measurements was used to obtain the final concentration, expressed in µg/mL.

Salivary concentrations of retinol-binding protein 4 (RBP4), β2-microglobulin, clusterin, cystatin C, interleukin (IL)-1β, IL-6, IL-8/CXCL8, IL-10, and tumor necrosis factor-α (TNF-α) were quantified using magnetic bead-based multiplex immunoassays from the MILLIPLEX^®^ MAP platform (MilliporeSigma, Burlington, MA, USA). RBP4, β2-microglobulin, clusterin, and cystatin C were measured using a customized four-analyte MILLIPLEX^®^ Human Kidney Injury Magnetic Bead Panel 6 kit (catalog no. HKI6MAG-99K-04), whereas IL-1β, IL-6, IL-8/CXCL8, IL-10, and TNF-α were quantified using a customized five-analyte MILLIPLEX^®^ Human Cytokine/Chemokine/Growth Factor Panel A kit (catalog no. HCYTA-60K-05). For the overnight protocols used, the manufacturer-reported minimum detectable concentrations were 0.013 ng/mL for RBP4, 0.043 ng/mL for β2-microglobulin, 1.853 ng/mL for clusterin, 0.404 ng/mL for cystatin C, 0.52 pg/mL for IL-1β, 0.14 pg/mL for IL-6, 0.52 pg/mL for IL-8/CXCL8, 0.91 pg/mL for IL-10, and 5.39 pg/mL for TNF-α. Salivary supernatants were thawed, gently mixed, and diluted 1:2 in the appropriate assay buffer. Samples, standards, and quality controls were analyzed in duplicate, and all samples were analyzed on a single 96-well plate for each panel to minimize inter-assay variability. Diluted samples were incubated with analyte-specific magnetic capture beads under agitation for 18 h at 4 °C for the kidney injury panel and at 2–8 °C for the cytokine panel. After washing, the beads were incubated with biotinylated detection antibodies for 1 h at room temperature, followed by incubation with streptavidin–phycoerythrin for 30 min under agitation at the same temperature. After the final washing step, the beads were resuspended in Drive Fluid and analyzed using an xMAP INTELLIFLEX^®^ instrument (Luminex Corporation, Austin, TX, USA). Median fluorescence intensity values were processed using Belysa^®^ software, version 1.2.1, and analyte concentrations were calculated from standard curves fitted using a five-parameter logistic regression model. Lower limits of quantification were determined using an accepted recovery range of 80–120% and a coefficient of variation limit of 20%, according to the predefined software settings. The run-specific lower limits of detection reported by the Belysa software were 0.210 pg/mL for IL-1β and 0.150 pg/mL for IL-10. The mean of the duplicate measurements was used as the final concentration for each analyte in each participant, and results were expressed in the assay-specific units reported by the acquisition software: ng/mL for RBP4, β2-microglobulin, clusterin, and cystatin C, and pg/mL for the cytokines.

### 2.6. Statistical Analysis

Statistical analyses were performed using jamovi software (version 2.7.34; The jamovi project, 2026). Data distribution was assessed using the Shapiro–Wilk test, and homogeneity of variances was evaluated using Levene’s test. Normally distributed continuous variables were summarized as mean ± standard deviation and compared between groups using the independent-samples Student’s *t*-test. When homogeneity of variances was not supported, Welch’s *t*-test was used. Non-normally distributed continuous variables were summarized as median [first–third quartiles] and compared using the Mann–Whitney U test. Categorical variables were presented as absolute and relative frequencies, whereas ordinal variables were additionally summarized as median [first–third quartiles] and compared between groups using the Mann–Whitney U test, as appropriate.

The primary analysis of interest was the adjusted comparison of the targeted panel of nine salivary biomarkers between women with high and low gingival bleeding burden. Because the study was exploratory, no individual biomarker was designated a priori as the sole primary outcome. ANCOVA models adjusted for maternal age and total salivary protein concentration constituted the primary analytical framework. The unadjusted between-group comparisons and the ANCOVA models additionally adjusted for visible biofilm percentage were considered complementary analyses.

For the adjusted analyses, salivary biomarker concentrations and total salivary protein concentration were log10-transformed to improve model fit and better satisfy distributional assumptions. Because zero concentrations were observed for IL-1β and IL-10, analyte-specific constants corresponding to one-half of the run-specific lower limits of detection reported by the Belysa software were added before log10 transformation: 0.105 pg/mL for IL-1β and 0.075 pg/mL for IL-10. Separate ANCOVA models were fitted for each salivary biomarker, with gingival bleeding group included as the fixed factor and maternal age and log10-transformed total salivary protein concentration included as covariates. Total salivary protein concentration was included to account for between-sample variation in overall salivary protein content, whereas maternal age was included a priori because of its potential association with maternal inflammatory profiles. Adjusted values were obtained from estimated marginal means on the log10-transformed scale and back-transformed to the original concentration scale. For IL-1β and IL-10, the corresponding constants were subtracted after back-transformation. Adjusted concentrations were reported as geometric means with 95% confidence intervals.

Because visible biofilm accumulation differed between groups and may contribute to the local inflammatory challenge, exploratory sensitivity ANCOVA models were additionally fitted by including visible biofilm percentage as a third covariate. These models assessed whether the group effects observed in the primary adjusted analyses were robust to further adjustment for biofilm burden.

Model assumptions were evaluated by inspection of residual diagnostic plots, including Q–Q plots, and by Shapiro–Wilk tests of residual normality. Homogeneity of variance between groups was assessed using Levene’s test applied to the log10-transformed dependent variables. Homogeneity of regression slopes was evaluated by testing group-by-covariate interaction terms. Where evidence of non-parallel slopes was observed, the robustness of the group contrast was further examined in interaction-inclusive models with continuous covariates centered at their sample means. Because Levene’s test indicated heterogeneity of variance for log10-transformed RBP4, the robustness of the RBP4 group effect was additionally evaluated using heteroscedasticity-consistent HC3 standard errors in both the primary and sensitivity models, while retaining their respective covariate specifications.

Partial eta squared (partial η^2^) was reported as a standardized measure of the adjusted group effect in each ANCOVA model. To address multiplicity across the nine salivary biomarkers, *p*-values were adjusted using the Benjamini–Hochberg false discovery rate procedure. The correction was applied separately to the nine unadjusted between-group comparisons, the nine primary ANCOVA group effects, and the nine sensitivity ANCOVA group effects because these represented distinct analytical families. Both raw and FDR-adjusted *p*-values were reported. All tests were two-sided, and FDR-adjusted *p*-values < 0.05 were considered statistically significant after correction for multiple testing.

A post hoc detectable-effect-size calculation was performed using G*Power software (version 3.1.9.7, Heinrich Heine University Düsseldorf, Düsseldorf, Germany) to quantify the magnitude of the adjusted group effect detectable with the available analytical sample. Because the group effect in the primary ANCOVA is equivalent to the incremental contribution of a binary group predictor in a linear regression model, the analysis was specified as a fixed multiple linear regression model testing R^2^ increase. The model included one tested predictor representing gingival bleeding group and two covariates, maternal age and log10-transformed total salivary protein concentration. With 24 participants, three predictors in the full model, α = 0.05, and 80% power, the minimum detectable effect size was Cohen’s f^2^ = 0.36. Thus, the available sample was capable of detecting relatively large adjusted group effects, whereas smaller effects may not have been detected. Accordingly, non-significant findings should not be interpreted as evidence of absence of association in the context of these exploratory biomarker comparisons.

## 3. Results

A total of 54 women met the eligibility criteria and underwent periodontal assessment. Of these, 12 had BOP ≥ 50% and were included in G1, 16 had BOP between 30% and <50% and were not included in the present comparative analysis, and 26 had BOP < 30%. From the latter group, 12 women were selected by computer-generated random sampling to form G2. The final analytical sample therefore comprised 24 participants, with 12 women in each group.

Table 1 shows the sociodemographic, systemic, oral hygiene, periodontal, and salivary characteristics of the study groups. No statistically significant between-group differences were observed in maternal age (*p* = 0.846), gravidity (*p* = 0.950), educational level (*p* = 0.975), monthly household income (*p* = 0.403), pre-pregnancy BMI (*p* = 0.349), gestational weight gain (*p* = 0.482), daily toothbrushing frequency (*p* = 0.381), daily interdental cleaning frequency (*p* = 0.055), salivary flow rate (*p* = 0.594), or total salivary protein concentration (*p* = 0.214). G1 presented higher visible biofilm (*p* = 0.005) and BOP (*p* < 0.001) than G2. Mean PPD and CAL were also slightly higher in G1 (*p* = 0.024 and *p* = 0.039, respectively), although both measures remained clinically low in both groups and were not indicative of destructive periodontal involvement.

Table 2 presents the unadjusted salivary biomarker comparisons. Before correction for multiple testing, between-group differences were observed for RBP4 (raw *p* < 0.001), β2-microglobulin (raw *p* = 0.041), clusterin (raw *p* < 0.001), IL-8 (raw *p* = 0.021), and IL-10 (raw *p* = 0.007). After FDR correction, differences remained statistically significant for RBP4 (FDR-adjusted *p* < 0.001), clusterin (FDR-adjusted *p* < 0.001), IL-8 (FDR-adjusted *p* = 0.047), and IL-10 (FDR-adjusted *p* = 0.021). G1 had higher unadjusted concentrations of RBP4, clusterin, and IL-8 and lower IL-10 concentrations than G2. The difference in β2-microglobulin did not remain statistically significant after FDR correction (FDR-adjusted *p* = 0.074).

The primary ANCOVA results are presented in Table 3. After adjustment for maternal age and total salivary protein concentration and correction for multiple testing, G1 showed higher RBP4 concentrations than G2 (raw *p* < 0.001; FDR-adjusted *p* = 0.004; partial η^2^ = 0.466). Clusterin was also higher in G1 (raw *p* = 0.001; FDR-adjusted *p* = 0.006; partial η^2^ = 0.413). Conversely, adjusted IL-10 concentrations were lower in G1 (raw *p* = 0.011; FDR-adjusted *p* = 0.032; partial η^2^ = 0.285). The adjusted group effects for β2-microglobulin (raw *p* = 0.042; FDR-adjusted *p* = 0.075; partial η^2^ = 0.191) and IL-8 (raw *p* = 0.038; FDR-adjusted *p* = 0.075; partial η^2^ = 0.198) did not remain statistically significant after FDR correction. No evidence of adjusted between-group differences was observed for cystatin C, IL-1β, IL-6, or TNF-α (all FDR-adjusted *p* > 0.05).

In the exploratory sensitivity analyses additionally adjusted for visible biofilm percentage, the group effect for RBP4 remained statistically significant after FDR correction (raw *p* = 0.003; FDR-adjusted *p* = 0.026; partial η^2^ = 0.381), whereas the group effect for IL-8 met the FDR-adjusted significance threshold (raw *p* = 0.010; FDR-adjusted *p* = 0.044; partial η^2^ = 0.303). Clusterin (raw *p* = 0.026; FDR-adjusted *p* = 0.078; partial η^2^ = 0.234) and IL-10 (raw *p* = 0.035; FDR-adjusted *p* = 0.078; partial η^2^ = 0.214) retained nominal between-group differences, but these did not remain statistically significant after FDR correction. The complete sensitivity analysis is presented in Supplementary Table S1.

**Figure 1:**
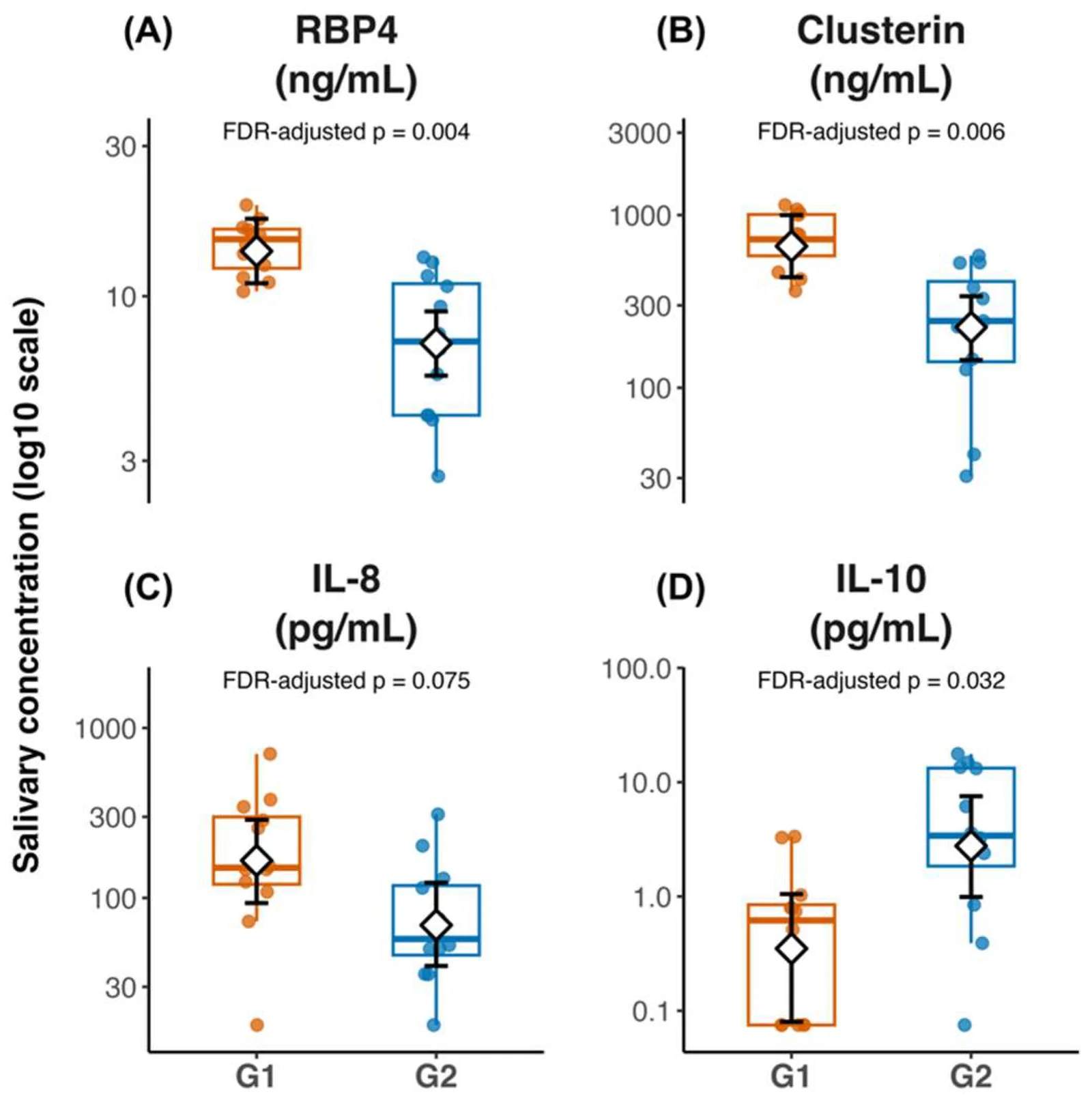
Observed distributions and primary-model adjusted geometric means (95% CIs) of salivary (**A**) RBP4, (**B**) clusterin, (**C**) IL-8, and (**D**) IL-10 according to gingival bleeding status. Points represent individual concentrations; boxplots show medians and interquartile ranges; and white diamonds with error bars represent adjusted geometric means and 95% CIs from ANCOVA models adjusted for maternal age and log10-transformed total salivary protein concentration. Displayed *p*-values were adjusted across the nine primary models using the Benjamini–Hochberg FDR procedure. IL-8 was included because it met the FDR-adjusted significance threshold in the sensitivity model additionally adjusted for visible biofilm (FDR-adjusted *p* = 0.044), although not in the primary model. Y-axes are logarithmic. G1, BOP ≥ 50%; G2, BOP < 30%. The orange color represents G1, and the blue color represents G2.

To illustrate data distributions and inter-individual variability alongside estimates obtained under a consistent model specification, Figure 1 presents RBP4, clusterin, IL-8, and IL-10, the four biomarkers that were statistically significant after FDR correction in at least one of the three analytical frameworks. Individual observations and boxplots show the unadjusted distributions, whereas all adjusted geometric means and 95% confidence intervals shown in the figure were derived from the primary ANCOVA models.

For RBP4, use of heteroscedasticity-consistent HC3 standard errors supported the robustness of the group effect in both the primary model (adjusted G1/G2 ratio = 1.97; 95% CI: 1.34–2.90; HC3-based *p* = 0.002) and the sensitivity model additionally adjusted for visible biofilm (adjusted G1/G2 ratio = 2.06; 95% CI: 1.11–3.83; HC3-based *p* = 0.025).

Exploratory interaction testing identified group-by-total-salivary-protein interactions for RBP4 (F_1,18_ = 4.863; *p* = 0.041; partial η^2^ = 0.213) and clusterin (F_1,18_ = 6.230; *p* = 0.022; partial η^2^ = 0.257), and a group-by-maternal-age interaction for IL-10 (F_1,18_ = 6.763; *p* = 0.018; partial η^2^ = 0.273). The same interaction terms remained nominally significant in the sensitivity models additionally adjusted for visible biofilm: RBP4, F_1,16_ = 4.954, *p* = 0.041, partial η^2^ = 0.236; clusterin, F_1,16_ = 5.452, *p* = 0.033, partial η^2^ = 0.254; and IL-10, F_1,16_ = 7.957, *p* = 0.012, partial η^2^ = 0.332. No group-by-visible-biofilm interactions were detected for these biomarkers (all *p* ≥ 0.276). In interaction-inclusive models with the continuous covariates centered at their sample means, the corresponding group contrasts remained in the same direction and were nominally significant in the primary models for RBP4 (Δlog_10_ = 0.287; 95% CI: 0.150–0.424; *p* < 0.001), clusterin (Δlog_10_ = 0.460; 95% CI: 0.227–0.692; *p* < 0.001), and IL-10 (Δlog_10_ = −0.825; 95% CI: −1.377 to −0.273; *p* = 0.006). Comparable contrasts were obtained in the sensitivity models for RBP4 (Δlog_10_ = 0.334; 95% CI: 0.149–0.519; *p* = 0.001), clusterin (Δlog_10_ = 0.383; 95% CI: 0.077–0.689; *p* = 0.017), and IL-10 (Δlog_10_ = −0.983; 95% CI: −1.710 to −0.256; *p* = 0.011).

## 4. Discussion

This study compared salivary proteins and inflammatory mediators in third-trimester pregnant women with contrasting gingival bleeding burdens, while excluding participants with clinical evidence of periodontitis or major systemic conditions that could influence periodontal inflammation or salivary biomarkers. No statistically significant between-group differences were observed in maternal age, gravidity, socioeconomic indicators, pre-pregnancy BMI, self-reported oral hygiene practices, unstimulated salivary flow rate, or total salivary protein concentration, strengthening the interpretation of differences in their salivary profiles. After FDR correction, the unadjusted comparisons identified higher RBP4, clusterin, and IL-8 and lower IL-10 concentrations in the high-bleeding group. In the primary analyses adjusted for maternal age and total salivary protein concentration, the group differences remained significant for RBP4, clusterin, and IL-10, whereas IL-8 retained only a nominal association. In the sensitivity analyses additionally adjusted for visible biofilm, RBP4 remained significant and the association with IL-8 became significant after FDR correction, whereas clusterin and IL-10 retained nominal, but not FDR-adjusted, associations. Collectively, these findings suggest that high gingival bleeding burden during pregnancy is associated with a salivary profile involving inflammatory/chemotactic, regulatory, and cellular stress-response signals. The persistence of the RBP4 and IL-8 associations after adjustment for visible biofilm further suggests that these differences were not fully explained by the greater biofilm burden in the high-bleeding group.

The grouping strategy used in this study was intended to increase clinical and biological contrast rather than to redefine gingivitis. According to the current classification, BOP at >30% of sites defines generalized gingivitis [30]. Nevertheless, BOP represents a continuous clinical measure, and women with values near this threshold may have overlapping inflammatory profiles, particularly during pregnancy, when gingival expression is influenced by interacting factors including biofilm accumulation, hormonal exposure, immune regulation, vascular changes, and oral microbiological shifts [5,35,36,37,38,39,40]. Women with BOP values from 30% to <50% were therefore excluded from the comparison, and the operational threshold of BOP ≥ 50%, previously used in salivary biomolecular studies of pregnant women with high gingival bleeding burden [20,21], was adopted to define the high-bleeding group. This strategy increased the contrast between the two ends of the bleeding spectrum and may have facilitated the identification of differences in salivary biomarkers. However, it also limits the generalizability of the findings, particularly to pregnant women with intermediate bleeding levels. Accordingly, the results should not be interpreted as supporting a distinct diagnostic threshold at BOP ≥ 50%, but as characterizing salivary profiles associated with contrasting gingival bleeding burdens in periodontitis-free pregnant women.

G1 had a higher percentage of sites with visible biofilm than G2. This finding is relevant because pregnancy gingivitis remains a dental biofilm-induced condition, although pregnancy-related hormonal, vascular, and immune changes may intensify the inflammatory response to a similar microbial challenge. Previous studies have shown that dental biofilm remains a major predictor of gingival inflammation during pregnancy, while hormonal and microbiological changes can modify its clinical expression [35,36,37,38,39,40]. However, the percentage of sites with visible biofilm reflects the clinical extent of plaque accumulation and does not characterize its microbial composition or inflammatory potential. Because microbial profiling and metagenomic analyses were not performed, we could not determine whether differences in biofilm composition also contributed to the contrasting bleeding burdens. Moreover, biofilm may act both as a confounding factor and as part of the pathway leading to gingival inflammation, roles that cannot be separated in a cross-sectional study. In this study, the primary models were therefore adjusted for maternal age and total salivary protein concentration, while visible biofilm was added in sensitivity analyses to examine how its inclusion affected the biomarker associations.

RBP4 and clusterin were the clearest non-cytokine findings in the primary analyses, showing the two largest adjusted group effects. Both were higher in G1 after FDR correction in the unadjusted and primary adjusted comparisons, but their associations responded differently to additional adjustment for visible biofilm. RBP4 remained significant in the sensitivity model, and the HC3 analyses yielded compatible estimates, making it the most consistent biomarker association in the study. Exploratory interactions with total salivary protein concentration were detected for both proteins. Although the conditional group contrasts at the mean total protein concentration remained directionally consistent and nominally significant, these interactions suggest that the magnitude of the group differences may vary with overall salivary protein content. Although primarily recognized as a retinol transport protein, RBP4 has been linked to periodontal inflammation and to inflammatory or metabolic complications of pregnancy [25,41,42]. Because pre-pregnancy BMI and gestational weight gain did not differ significantly between groups and major metabolic conditions were excluded, higher salivary RBP4 in G1 should not be interpreted as evidence of systemic metabolic dysfunction. Its persistence after biofilm adjustment indicates that the association was not fully explained by the greater clinical extent of biofilm, although the source and function of salivary RBP4 remain unclear. Clusterin, by contrast, retained a nominal association after biofilm adjustment but did not remain statistically significant after FDR correction. As a stress-responsive molecular chaperone involved in proteostasis, oxidative stress, and tissue protection, its increase may reflect a compensatory response to the greater inflammatory challenge rather than direct evidence of tissue damage [27]. A previous salivary proteomic study reported lower clusterin concentrations in gingivitis and periodontitis than in periodontal health [43], suggesting that its direction of change may depend on disease stage and biological context. Clusterin should therefore be considered a plausible but less stable component of the salivary profile associated with high gingival bleeding.

The cytokine findings in this study pointed to differences in inflammatory regulation rather than to a generalized increase in pro-inflammatory mediators. IL-10 was lower in G1 after FDR correction in both the unadjusted comparison and the primary adjusted model. IL-10 limits excessive cytokine production and helps contain tissue-damaging immune responses [24,44,45]. Its lower salivary concentration in G1 is therefore compatible with less prominent anti-inflammatory counter-regulation in women with high gingival bleeding, but it does not demonstrate an IL-10 deficiency or a causal role in the clinical phenotype. After inclusion of visible biofilm, the direction of the group difference and the nominal association were retained, although the result no longer met the FDR-adjusted significance threshold. This attenuation may indicate that part of the IL-10 difference was related to the contrasting biofilm burden, but reduced precision after adding another covariate to a small-sample model is also possible. The exploratory group-by-age interaction observed for IL-10 warrants caution, although the group contrast estimated at the mean maternal age remained comparable to that of the primary model.

IL-8 provided a complementary chemotactic signal. Its concentration was higher in G1 after FDR correction in the unadjusted comparison, whereas the group effect in the primary model was nominally significant but did not remain so after FDR correction. Because G1 had more extensive visible biofilm, adjustment would be expected to attenuate the IL-8 group effect if greater biofilm accumulation explained the higher concentrations. Instead, the estimated group effect increased and met the FDR-adjusted significance threshold, indicating that G1 had higher predicted IL-8 concentrations than G2 at the same modeled percentage of sites with visible biofilm. This pattern is compatible with a stronger chemotactic response relative to the clinical extent of biofilm, rather than an IL-8 difference driven solely by greater biofilm accumulation. It should not, however, be interpreted as independent of the microbial challenge, because visible biofilm does not characterize microbial biomass, composition, or inflammatory potential. IL-8 is a major chemokine for neutrophil recruitment and activation at biofilm-challenged sites, and pregnancy-gingivitis proteomic studies have similarly identified neutrophil-mediated responses and extracellular-trap-related pathways [22,23,46,47]. Relatively higher salivary IL-8 in G1 is compatible with stronger neutrophil recruitment and may help explain the more pronounced bleeding response in G1 and, if sustained, could contribute to proteolytic and oxidative tissue injury. However, because all participants were free of periodontitis and no longitudinal progression was assessed, greater susceptibility to future periodontal breakdown remains a hypothesis. Considered together, higher IL-8 and lower IL-10 are compatible with a salivary environment characterized by stronger chemotactic signaling and less evident anti-inflammatory regulation, although the two associations showed different stability across the adjusted models.

The absence of group differences in IL-1β, IL-6, and TNF-α provides an important counterpoint to the IL-8 and IL-10 findings. Although these cytokines have established roles in periodontal inflammation and tissue destruction [22,23], their concentrations in whole saliva may not closely reflect localized production in gingival tissues or gingival crevicular fluid because whole saliva integrates contributions from multiple oral and systemic sources [48,49]. Moreover, the participants were free of periodontitis and had clinically low probing depth and attachment-level measurements, which may partly explain the absence of a broader cytokine pattern associated with destructive periodontal inflammation. Differences in cytokine kinetics and salivary dilution, together with pregnancy-related immune modulation, may also have contributed [5,35,36,37,38,39,40,48,49]. These non-significant results do not exclude local involvement of IL-1β, IL-6, or TNF-α, particularly because the sample was primarily capable of detecting relatively large effects. β2-microglobulin showed nominal differences but did not remain significant after FDR correction, despite previous evidence of its increase in saliva from pregnant women with excessive gingival bleeding and in gingival crevicular fluid from patients with periodontal diseases [21,26]. No consistent group difference was identified for cystatin C. The resulting selective profile is consistent with previous salivary proteomic studies showing that pregnancy-associated gingival inflammation involves coordinated changes in neutrophil responses, antimicrobial defense, oxidative stress, and tissue-protective pathways rather than a uniform increase in classical pro-inflammatory cytokines [20,21,46,47]. Because these studies used broader proteomic approaches, the convergence should be interpreted at the pathway level rather than as direct replication of individual biomarkers.

The findings should be considered in light of the exploratory, cross-sectional design and limited sample size. Nine biomarkers were evaluated without designating an individual analyte as the sole primary outcome, increasing the possibility of Type I error through multiple testing. Although FDR correction was applied separately to the three analytical families, the possibility of chance findings cannot be completely eliminated. Accordingly, nominal associations that did not remain significant after correction and the exploratory interaction findings should be regarded as hypothesis-generating. With 24 participants, the study was primarily capable of detecting relatively large adjusted effects; smaller associations may have gone undetected, and the estimated effect magnitudes may be imprecise. The cross-sectional assessment does not establish temporal sequence, causality, or subsequent periodontal progression. In addition, the comparison of Brazilian women in the third trimester at the two ends of the gingival bleeding spectrum limits generalization to women with intermediate bleeding, other stages of pregnancy, and broader populations. Because participants with periodontitis were excluded, the findings should not be extrapolated directly to destructive periodontal disease.

The main contribution of this study is the characterization of a selective salivary profile in pregnant women with high gingival bleeding but without periodontitis. Clinically, the findings indicate that extensive gingival bleeding may be accompanied by host-response differences not fully accounted for by the clinical extent of visible biofilm. Saliva is suitable for repeated assessment during pregnancy because its collection is non-invasive and causes minimal discomfort. These results, however, do not support replacing bleeding on probing, biofilm recording, or a complete periodontal examination.

Clinical translation of salivary biomarkers remains challenging. Salivary composition varies with flow rate, circadian rhythms, collection and processing procedures, and contributions from local and systemic sources; inadequate study design, sample size, and control selection may also produce findings that are difficult to reproduce [48,49,50]. The present study was not designed to establish diagnostic thresholds or predictive accuracy. Larger longitudinal studies should include women across the full spectrum of gingival bleeding, evaluate repeated samples throughout pregnancy and postpartum, and determine whether the observed profile precedes, accompanies, or resolves with changes in periodontal inflammation. Combining salivary biomarkers with microbial profiling and parallel measurements in gingival crevicular fluid or blood may also help clarify their biological origin and specificity. Until such validation is available, RBP4, clusterin, IL-8, and IL-10 should be regarded as components of an associated salivary profile rather than diagnostic or prognostic biomarkers. Their clinical value will depend on demonstrating reproducibility, temporal stability, and incremental information beyond conventional periodontal assessment. Overall, high gingival bleeding burden in late pregnancy was associated with a selective salivary profile involving inflammatory, regulatory, and stress-response signals. These findings characterize an association rather than a causal or prognostic relationship and do not establish future periodontal tissue breakdown.

## Figures and Tables

**Table 1:** Sociodemographic, systemic, oral hygiene, periodontal, and salivary characteristics according to gingival bleeding status.

	G1 (*n* = 12) Mean ± SD Median [Q1–Q3]	G2 (*n* = 12) Mean ± SD Median [Q1–Q3]	*p*
Age (years)	27.20 ± 6.12	26.60 ± 8.31	0.846 *
Gravidity	1.50 [1–3]	1.50 [1–2.25]	0.950 ^†^
Educational level–n (%) No formal education (0) Incomplete primary education (1) Completed primary education (2) Incomplete secondary education (3) Completed secondary education (4) Incomplete higher education (5) Completed higher education (6)	4 [3.75–4] 0 (0) 2 (16.70) 0 (0) 1 (8.30) 7 (58.30) 0 (0) 2 (16.70)	4 [3–4.50] 0 (0) 1 (8.30) 1 (8.30) 2 (16.70) 5 (41.70) 0 (0) 3 (25)	0.975 ^†^
Monthly income–n (%) Up to one MW (1) 1–2 MW (2) 2–3 MW (3) 3–4 MW (4) 4–5 MW (5) More than 5 MW (6)	2 [2–3] 1 (8.30) 6 (50) 4 (33.40) 0 (0) 0 (0) 1 (8.30)	3 [1.75–4.25] 3 (25) 1 (8.30) 4 (33.40) 1 (8.30) 1 (8.30) 2 (16.70)	0.403 ^†^
Pre-pregnancy BMI (kg/m^2^)	26.50 ± 3.84	28.60 ± 6.51	0.349 ^‡^
Gestational weight gain (kg)	10.30 ± 6.00	8.74 ± 4.46	0.482 *
Daily toothbrushing	3 [2–3]	3 [3–3]	0.381 ^†^
Daily interdental cleaning	0 [0–0.25]	1 [0–2]	0.055 ^†^
Visible biofilm (%)	67.50 ± 21.10	41.30 ± 19.80	0.005 *
BOP (%)	55.10 ± 6.06	24.80 ± 5.70	<0.001 *
PPD (mm)	2.06 [2.04–2.16]	2.03 [1.98–2.04]	**0.024 ^†^**
CAL (mm)	2.09 [2.04–2.15]	2.03 [1.98–2.05]	**0.039 ^†^**
Salivary flow (mL/min)	0.59 ± 0.05	0.58 ± 0.04	0.594 *
Total salivary protein concentration (µg/mL)	1275 [829–1380]	1379 [1086–2052]	0.214 ^†^

Between-group comparisons were performed using independent-samples Student’s *t*-test (*), Mann–Whitney U test (^†^), or Welch’s *t*-test (^‡^), as appropriate. G1, high gingival bleeding group (BOP ≥ 50%); G2, low gingival bleeding group (BOP < 30%); SD, standard deviation; Q1, first quartile; Q3, third quartile; MW, minimum wage; BMI, body mass index; BOP, bleeding on probing; PPD, probing pocket depth; CAL, clinical attachment level. Bold values indicate statistical significance at *p* < 0.05.

**Table 2:** Unadjusted salivary concentrations of proteins and inflammatory mediators according to gingival bleeding status.

Salivary Biomarker	Unadjusted Mean ± SD Unadjusted Median [Q1–Q3]			
	G1 (*n* = 12)	G2 (*n* = 12)	Raw *p*	FDR-Adjusted *p*
RBP4 (ng/mL)	14.60 ± 2.82	7.74 ± 3.73	<0.001 *	**<0.001**
β2-microglobulin (ng/mL)	184 ± 61.70	124 ± 73.40	0.041 *	0.074
Clusterin (ng/mL)	753 ± 264	284 ± 189	<0.001 *	**<0.001**
Cystatin C (ng/mL)	36.40 ± 6.16	39.60 ± 9.20	0.337 *	0.433
IL-1β (pg/mL)	18.20 ± 17.40	8.12 ± 6.08	0.079 ^†^	0.118
IL-6 (pg/mL)	3.28 [2.21–4.81]	3.16 [2.32–4.18]	0.977 ^‡^	0.977
IL-8 (pg/mL)	151 [101–301]	57.60 [50.20–118]	0.021 ^‡^	**0.047**
IL-10 (pg/mL)	0.62 [0–0.85]	3.41 [2–13.30]	0.007 ^‡^	**0.021**
TNF-α (pg/mL)	13.60 [9.25–30.40]	15.10 [9.11–18.30]	0.590 ^‡^	0.664

RBP4, β2-microglobulin, clusterin, and cystatin C concentrations are expressed in ng/mL, whereas cytokine concentrations are expressed in pg/mL. Values are presented as mean ± standard deviation or median [first–third quartiles], according to data distribution. Between-group comparisons were performed using the independent-samples Student’s *t*-test (*), Welch’s *t*-test (^†^), or Mann–Whitney U test (^‡^), as appropriate. The Benjamini–Hochberg procedure was applied across the nine biomarker comparisons to control the false discovery rate. G1, high gingival bleeding group (BOP ≥ 50%); G2, low gingival bleeding group (BOP < 30%); BOP, bleeding on probing; SD, standard deviation; Q1, first quartile; Q3, third quartile; FDR, false discovery rate. Bold FDR-adjusted *p*-values indicate statistical significance after correction for multiple testing at *p* < 0.05.

**Table 3:** Salivary concentrations of proteins and inflammatory mediators according to gingival bleeding status, adjusted for maternal age and total salivary protein concentration.

Salivary Biomarker	Adjusted Geometric Mean (95% CI)				
	G1 (*n* = 12)	G2 (*n* = 12)	Raw *p*	FDR-Adjusted *p*	Partial η^2^
RBP4 (ng/mL)	13.90 (10.99–17.62)	7.08 (5.59–8.95)	<0.001	**0.004**	0.466
β2-microglobulin (ng/mL)	169.82 (125.89–229.09)	109.65 (81.28–147.91)	0.042	0.075	0.191
Clusterin (ng/mL)	660.69 (436.52–1000.00)	223.87 (144.54–338.84)	0.001	**0.006**	0.413
Cystatin C (ng/mL)	35.48 (30.90–40.74)	38.90 (33.88–44.67)	0.372	0.558	0.040
IL-1β (pg/mL)	6.25 (1.78–21.27)	3.84 (1.06–13.08)	0.574	0.646	0.016
IL-6 (pg/mL)	3.34 (2.06–5.41)	3.18 (1.96–5.14)	0.879	0.879	0.001
IL-8 (pg/mL)	165.96 (93.33–288.40)	69.18 (39.81–123.03)	0.038	0.075	0.198
IL-10 (pg/mL)	0.35 (0.08–1.05)	2.77 (0.99–7.53)	0.011	**0.032**	0.285
TNF-α (pg/mL)	16.60 (10.47–26.30)	13.49 (8.47–21.38)	0.511	0.646	0.022

RBP4, β2-microglobulin, clusterin, and cystatin C concentrations are expressed in ng/mL, whereas cytokine concentrations are expressed in pg/mL. Adjusted values are geometric means with 95% confidence intervals derived from estimated marginal means of log10-transformed ANCOVA models and back-transformed to the original concentration scale. Separate models were fitted for each biomarker, with gingival bleeding group included as the fixed factor and maternal age and log10-transformed total salivary protein concentration included as covariates. For IL-1β and IL-10, constants of 0.105 pg/mL and 0.075 pg/mL, respectively, were added before log10 transformation because zero concentrations were present; the corresponding constants were subtracted after back-transformation. Partial eta squared (partial η^2^) represents the standardized effect size for the adjusted group effect. The Benjamini–Hochberg procedure was applied across the nine adjusted group comparisons to control the false discovery rate. G1, high gingival bleeding group (BOP ≥ 50%); G2, low gingival bleeding group (BOP < 30%); BOP, bleeding on probing; CI, confidence interval; ANCOVA, analysis of covariance; FDR, false discovery rate. Bold FDR-adjusted *p*-values indicate statistical significance after correction for multiple testing at *p* < 0.05.

## Data Availability

The data presented in this study are available from the corresponding author upon reasonable request. The data are not publicly available to protect participant confidentiality.

## Supplementary Materials

The following supporting information can be downloaded at https://www.mdpi.com/article/10.3390/life16091423/s1, Table S1. Exploratory sensitivity ANCOVA models for salivary proteins and inflammatory mediators according to gingival bleeding status, additionally adjusted for visible biofilm percentage.
