# Supplementary material for: High Gingival Bleeding Burden in Late Pregnancy Is Associated with a Salivary Profile of Inflammatory, Regulatory, and Stress-Response Signals
Source: Life (Basel). 2026 Aug 27;16(9):1423. doi: 10.3390/life16091423 (PMC13608516; doi:10.3390/life16091423)
Supplement: Supplementary file 1 [file life-16-01423-s001.zip › life-4487813-supplementary.pdf]

Table S1. Exploratory sensitivity ANCOVA models for salivary proteins and inflammatory mediators according to gingival bleeding status, additionally adjusted for visible biofilm percentage.

Salivary biomarker	Adjusted geometric mean (95% CI)				
	G1 (n = 12)	G2 (n = 12)	Raw p	FDR-adjusted p	Partial η²
**RBP4 (ng/mL)**	14.23 (10.78–18.79)	6.91 (5.24–9.12)	0.003	**0.026**	0.381
**β2-microglobulin (ng/mL)**	158.24 (112.46–222.68)	117.34 (83.39–165.12)	0.264	0.396	0.065
**Clusterin (ng/mL)**	600.23 (367.61–980.03)	244.83 (149.95–399.76)	0.026	0.078	0.234
**Cystatin C (ng/mL)**	35.32 (30.15–41.36)	39.22 (33.48–45.93)	0.394	0.452	0.038
**IL-1β (pg/mL)**	7.85 (1.81–32.85)	3.04 (0.65–12.95)	0.402	0.452	0.037
**IL-6 (pg/mL)**	3.14 (1.78–5.54)	3.38 (1.92–5.96)	0.866	0.866	0.002
**IL-8 (pg/mL)**	210.35 (113.53–389.73)	54.63 (29.49–101.22)	0.010	**0.044**	0.303
**IL-10 (pg/mL)**	0.33 (0.05–1.21)	2.92 (0.86–9.52)	0.035	0.078	0.214
**TNF-α (pg/mL)**	19.24 (11.43–32.41)	11.52 (6.84–19.39)	0.210	0.379	0.081

RBP4, β2-microglobulin, clusterin, and cystatin C concentrations are expressed in ng/mL, whereas cytokine concentrations are expressed in pg/mL. Adjusted values are geometric means with 95% confidence intervals derived from estimated marginal means of log10-transformed ANCOVA models and back-transformed to the original concentration scale. Separate models were fitted for each biomarker, with gingival bleeding group included as the fixed factor and maternal age, log10-transformed total salivary protein concentration, and visible biofilm percentage included as covariates. For IL-1β and IL-10, constants of 0.105 pg/mL and 0.075 pg/mL, respectively, were added before log10 transformation because zero concentrations were present; the corresponding constants were subtracted after back-transformation. Partial eta squared (partial η^2) represents the standardized effect size for the adjusted group effect. The Benjamini–Hochberg procedure was applied across the nine sensitivity ANCOVA group effects to control the false discovery rate. G1, high gingival bleeding group (BOP ≥50%); G2, low gingival bleeding group (BOP <30%); BOP, bleeding on probing; CI, confidence interval; ANCOVA, analysis of covariance; FDR, false discovery rate. Bold FDR-adjusted p-values indicate statistical significance after correction for multiple testing at $p < 0.05$.
